# Colour discrimination thresholds vary throughout colour space in a reef fish (*Rhinecanthus aculeatus*)

**DOI:** 10.1242/jeb.243533

**Published:** 2022-04-11

**Authors:** Naomi F. Green, Emily Guevara, Daniel C. Osorio, John A. Endler, N. Justin Marshall, Misha Vorobyev, Karen L. Cheney

**Affiliations:** 1 School of Biological Sciences, The University of Queensland, Brisbane, Queensland 4072, Australia; 2 Queensland Brain Institute, The University of Queensland, Brisbane, Queensland 4072, Australia; 3 School of Life Sciences, The University of Sussex, Falmer, Brighton BN1 9QG, UK; 4 Centre for Integrative Ecology, School of Life and Environmental Sciences, Deakin University, Victoria 3216, Australia; 5 Department of Optometry and Vision Science, The University of Auckland, Auckland 1142, New Zealand

**Keywords:** Visual ecology, Colour vision, Receptor noise limited model, Colour thresholds, Discrimination thresholds

## Abstract

Animals use colour vision in a range of behaviours. Visual performance is limited by thresholds, which are set by noise in photoreceptors and subsequent neural processing. The receptor noise limited (RNL) model of colour discrimination is widely used for modelling colour vision and accounts well for experimental data from many species. In one of the most comprehensive tests yet of colour discrimination in a non-human species, we used Ishihara-style stimulus patterns to examine thresholds for 21 directions at five locations in colour space for the fish *Rhinecanthus aculeatus*. Thresholds matched RNL model predictions most closely for stimuli near the achromatic point, but exceeded predictions (indicating a decline in sensitivity) with distance from this point. Thresholds were also usually higher for saturation than for hue differences. These changes in colour threshold with colour space location and direction may give insight into photoreceptor non-linearities and post-receptoral mechanisms of colour vision in fish. Our results highlight the need for a cautious interpretation of the RNL model – especially for modelling colours that differ from one another in saturation (rather than hue), and for highly saturated colours distant from the achromatic point in colour space.

## Abstract (precis)

**Summary:** A comparison of thresholds for colour sets with similar orientations in colour space but located at different distances from the adapted achromatic point.

## INTRODUCTION

Animals use colour for a range of tasks, especially foraging, communication and reproduction. Visual photoreceptors provide the interface between the brain and the world, and ultimately limit what an animal can see. The number of spectral receptor types ranges from one to 15 in different animal eyes and they cover different parts of the UV and human-visible spectrum (from approximately 300 to 700 nm; e.g. Osorio and Vorobyev, 2005). As colour vision is based on comparison of the signals from two or more spectral receptor types, species differences in photoreceptor spectral sensitivities will cause differences in colour perception, but neural processing beyond the photoreceptors also needs to be taken into account if we are to understand how non-human species see colour. This study evaluates the contribution of these post-receptoral mechanisms for a colour discrimination task in a reef fish, *Rhinecanthus aculeatus*. This was done by comparing experimental colour discrimination thresholds with those predicted by the widely used receptor noise limited (RNL) model, which assumes that these thresholds are set by photoreceptors. Discrepancies between the fishes' observed performance and model predictions give insight into neural mechanisms of colour vision, as well as limitations of the RNL model.

Modelling of colour vision for an animal starts with estimation of its photoreceptor responses to a coloured object, which are specified by the receptors' spectral sensitivities and the illumination and reflectance spectra. Colours can be represented as points in a chromaticity diagram (or colour space) whose axes are given by estimated responses (e.g. photon absorptions per receptor per second) of the spectral receptor types (Kelber et al., 2003). For an eye with *n* receptor types, the primary colour space has *n*–1 dimensions, yielding a colour triangle or a hexagon for trichromatic vision, and a tetrahedron for tetrachromatic vision (Kelber et al., 2003; Chittka, 1992).

**Fig. 1.**
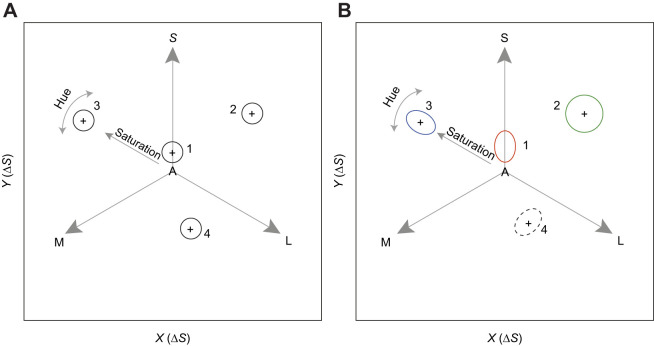
**Loci of discrimination thresholds for different models of colour vision in a chromaticity diagram whose axes are given in equations in** **Pike (2012****).** Vectors S, M and L represent short-, medium- and long-wavelength photoreceptor responses, respectively (for a trichromatic species). The A in the centre represents the adapting background. Ellipses illustrate the locus of the discrimination threshold (Δ*S*) from its reference colour (+). (A) The receptor noise limited (RNL) model predicts circular loci of fixed radius (Δ*S*=1) across colour space (black circles). (B) Possible causes of departures from the RNL model predictions include: (1) noise in one or more photoreceptor channels exceeds that predicted from the relative number of the S, M and L receptors [here, loci are elongated in the S direction (red ellipse) attributable to noise in the S-cone mechanism exceeding that predicted by the relative cone abundance]; (2) thresholds increase with colour saturation from the achromatic point (green circle); (3) thresholds for hue may be smaller than those for saturation (blue ellipse); and (4) elliptical thresholds may result from compression or expansion of some areas of colour space owing to opponent mechanisms (dashed ellipse); however, this was not directly addressed in this study because the RNL model assumes no or unspecified opponent calculations. These examples of deviations from RNL model predictions are not exhaustive, and assume that noise in each cone mechanism is proportional to the mean response (i.e. Weber's law holds).

Colour spaces can be transformed using either behavioural or neurophysiological data so that the distance between points corresponds to the colour difference perceived by a given viewer (Kelber et al., 2003; Vorobyev and Brandt, 1997; Backhaus, 1991). There has been a longstanding interest in developing a uniform colour space for humans, where the discrimination threshold is a fixed distance throughout the space (Fig. 1). However, in practice, commonly used spaces such as CIE Lab and CIE LUV are not uniform; instead, threshold loci are often elliptical and vary in size with their location (Danilova and Mollon, 2016; Wyszecki and Stiles, 1982; Judd, 1968; MacAdam, 1942). The difficulty in finding a uniform colour space reflects the complex non-linear nature of colour processing involving a multistage neural pathway running from the retina through the brain. The comparison of colour thresholds throughout colour space can therefore offer insights into colour processing.

Most studies of colour discrimination in animals have relied on monochromatic lights, limiting them to examinations of hue discrimination close to the monochromatic locus (Goldsmith et al., 1981; Neumeyer, 1986; White et al., 1994); few studies have examined colour discrimination thresholds throughout colour space in non-human animals (but see Sibeaux et al., 2019; Champ et al., 2016, 2014; Olsson et al., 2015). Theoretical vision models are widely used to predict colour thresholds in animals (Kelber et al., 2003; Renoult et al., 2017), including the RNL model of colour discrimination, which postulates that thresholds are set by chromatic opponent mechanisms whose performance is limited by noise originating in the photoreceptors (Vorobyev and Osorio, 1998; Vorobyev et al., 2001). This model requires a noise value in each receptor mechanism, which can be based on direct electrophysiological measurements, or estimated (Vorobyev and Osorio, 1998; Vorobyev et al., 2001). Often is it assumed that receptor noise has a fixed standard deviation relative to the mean response for each spectral type of receptor in daylight (consistent with Weber's law), and that the relative noise levels in different spectral mechanisms depend on the relative abundance of each receptor type (Vorobyev and Osorio, 1998).

To the limits of experimental accuracy, the RNL model predicts thresholds for detection of minimally saturated colours on grey adapting backgrounds in a range of animals including butterflies, bees, reptiles, birds and fish (Champ et al., 2016; Olsson et al., 2015; de Ibarra et al., 2014; Fleishman et al., 2016; Arikawa, 2017). For humans, the RNL model prediction is good but not exact (Vorobyev and Osorio, 1998). However, in guppies, there is evidence that colour thresholds vary depending on the direction of the colour change near the achromatic point (Sibeaux et al., 2019). In blue tits, the sensitivity of colour detection thresholds was reduced when objects were placed against highly saturated coloured backgrounds (Silvasti et al., 2021). The RNL model is used widely in studies on visual ecology, including camouflage, mimicry, sexual signalling, territoriality, foraging, and the evolution of animal colouration and perceptual systems (e.g. Tedore and Nilsson, 2021; Cheney and Marshall, 2009; Siddiqi et al., 2004; Hastad et al., 2005; Stuart-Fox et al., 2003), and deviations from model predictions may alter overall conclusions derived from such studies.

**Fig. 2.**
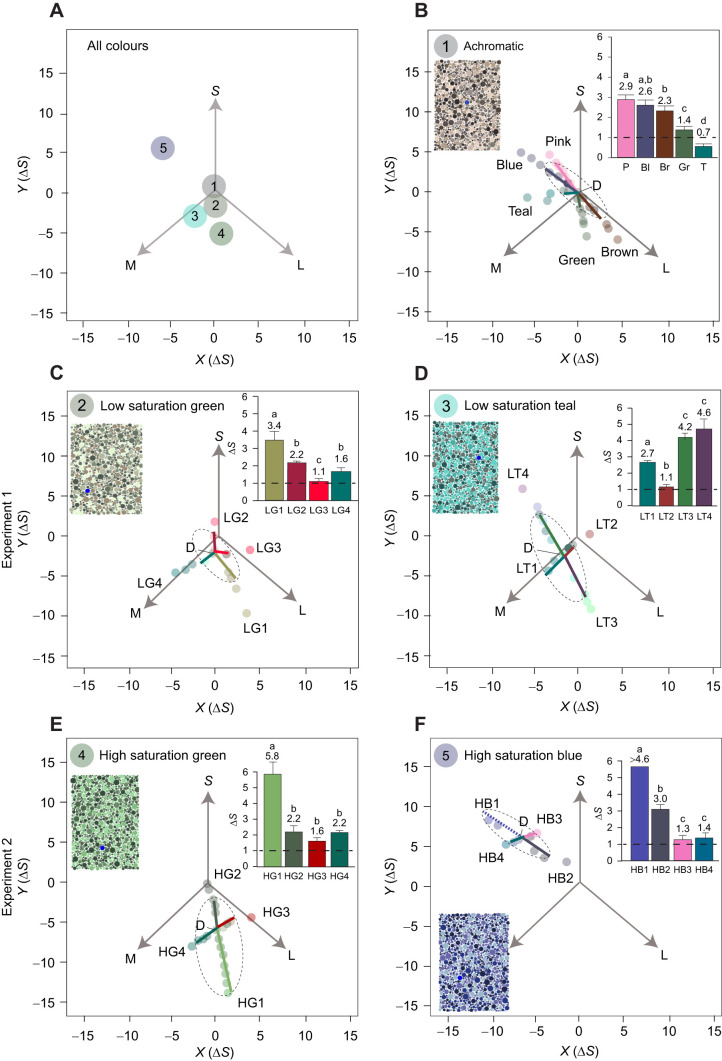
**Target colours and distractors that were used to examine colour thresholds of *Rhinecanthus aculeatus*.** (A) The relative position of each of the distractor sets in RNL space (Pike, 2012) using *w*_S_=0.07, *w*_M_=0.05 and *w*_L_=0.05: (1) achromatic (as per Cheney et al., 2019); (2) low saturation green; (3) low saturation teal; (4) high saturation green; and (5) high saturation blue. (B–F) Examples of each distractor set as an inset with a blue dot providing an example of a target dot which fish were required to discriminate from the distractor dots and peck to receive a food reward. The location of the distractors is identified by a ‘D’ (distractors only varied in luminance and are therefore co-located in RNL space). The remaining coloured circles in each figure (which produce two approximately perpendicular lines) show the colours of the target dots (with opacity set at 40% for illustrative purposes), which fish had to find and discriminate from distractor dots. Vectors S, M and L, represent the relative photoreceptor stimulation of the short-, medium- and long-wavelength photoreceptors, respectively. Behaviourally measured colour thresholds are shown as lines from the location of distractors to the threshold value in the direction tested. The threshold for HB1 is shown as a dashed line as fish did not reach 50% success for any of the colours tested in this set; therefore, this threshold would be higher than shown here. Unfortunately, we could not test more colours further away from the distractor dots in this direction due to limitations of our printer. We have also added ellipses (dashed lines) around the thresholds for each distractor set. To facilitate side-by-side comparison, thresholds are also shown as bars that indicate the means±s.e.m. (inset) and numerical values above bars (mean). Different lowercase letters in the same graph indicate a significant difference (*P*<0.05). Dashed lines on bar plots are predictions from the RNL model with discrimination thresholds Δ*S*=1.0.

Here, we compare behavioral thresholds across colour space for a coral reef fish (*R. aculeatus*) with those predicted by the RNL model. We report results from four directions at each of four locations in colour space, and compare them with five directions close to the achromatic point from a previous study using the same methodology (Cheney et al., 2019). Fig. 1 outlines some ways in which colour thresholds depart from RNL model predictions across colour space, including non-linear responses from photoreceptors or retinal neurons (Chittka, 1992; Laughlin, 1981), spectral opponent mechanisms (Chittka, 1992; Hurvich and Jameson, 1957), or higher-level processing including memory (Dyer and Neumeyer, 2005), learning (Avargues-Weber et al., 2010) and colour categorisation (Jones et al., 2001; Caves et al., 2018). To measure colour thresholds, we use an Ishihara-style test (Cheney et al., 2019), where the fish is challenged to find an ‘odd-one-out’ target dot within an array of distractor dots (Fig. 2).

## MATERIALS AND METHODS

### Study species

The triggerfish *Rhinecanthus aculeatus* (Linnaeus 1758) is a generalist omnivore, feeding primarily on molluscs and crustaceans on sub-tidal coral reef flats throughout the Indo-Pacific, which performs well in behavioral experiments (Cheney et al., 2019). For this study, fish ranged from 10 to 26 cm total length; sex could not be determined. They were obtained from an aquarium supplier (Cairns Marine Pty Ltd, Cairns, Australia) or collected around Lizard Island, Great Barrier Reef, Australia, using hand nets on snorkel (QLD General Fisheries Permit 183990; Great Barrier Reef Marine Park Authority Permit G16/38497.1). Fish were housed at the University of Queensland in separate tanks according to size (60×40×30 cm to 100×50×50 cm) and allowed to acclimatise for at least 3 to 8 weeks before experiments began. We conducted two experiments: experiment 1 was carried out between November 2016 and January 2017 (*n*=9 fish) and experiment 2 between July and September 2016 (*n*=6 fish), with one fish used in both experiments (fish B). A small number of these fish had been previously tested with the achromatic distractors in February–April 2016 (Cheney et al., 2019) (experiment 1: fish B, N and O; experiment 2: fish B, C and E). Some fish were reused in different experiments to reduce the total number of individuals used in this study. To ensure that factors such as previous experience or training duration did not impact their overall performance, we statistically tested for differences in thresholds between new and reused animals, which were non-significant (see ‘Statistical analysis’ section). Experiments were approved by the University of Queensland's Animal Ethics Committee (SBS/111/14/ARC).

### Experimental setup

Opaque partitions with doors were placed in the centre of the tank, which allowed fish to be excluded from one end while stimuli were placed in position. Ishihara-style printed stimuli were first layered on top of a second sheet of plain paper to prevent the colour behind the paper affecting the colour of dots when submerged in water. Dots are not affected by water because laser-printed dots are melted plastic. A small piece of squid was placed beneath the target dot between the two layers of paper. The two sheets were then secured on top of a PVC board using an elastic band at each end. Finally, the prepared stimuli were placed horizontally on the substrate in the testing arena. During training (protocol described in Cheney et al., 2019), fish were taught to locate target dots that differed from the colour of the distractors by a colour distance, Δ*S*, of ∼7, which were easily detected by the fish.

### Training protocol

During training, fish were taught to locate target dots that differed from the colour of the distractors by a Δ*S* of 4–7, which were easily detected by the fish. Training colours included purple, teal, pink, red, blue and green, which were presented in a randomised sequence to prevent fish learning to associate a particular colour with food. Training began with a conspicuous target dot set against: (1) a solid achromatic (grey) background; (2) an Ishihara-style stimulus featuring achromatic distractor dots; and, finally, (3) an Ishihara-style stimulus featuring coloured distractors appropriate to the experiment (experiment 1: the low saturation green and low saturation teal distractor sets: Fig. 2C,D; experiment 2: the high saturation green and high saturation blue distractor sets: Fig. 2E,F). Fish progressed from one training level to the next once they had achieved the learning criterion of 90% correct pecks over three sequential sessions. Fish readily performed the behavior, regardless of the colour of the distractors. After training, we determined whether fish could locate the target dots using olfactory cues alone. To do this, we conducted control trials in which food was placed under a random distractor dot. However, during these trials, fish only located the food on 3 of 48 trials (6% success), suggesting that fish primarily used visual cues to locate the food.

### Experimental trials

Each experimental trial began once the partition was opened and fish entered the testing arena. As per Cheney et al. (2019), we recorded: (1) whether the fish was successful in pecking the target dot (usually only once) to access the food reward within 30 s of entering the test arena; (2) if so, the time taken from entry to pecking at the target (latency to find the dot); and (3) the number of distractor dots that were pecked incorrectly before the target was pecked (Table 1). Interestingly, the fish always pecked directly on a dot and not in between dots or elsewhere on the paper. After the target dot had been pecked or 30 s had elapsed, the fish were gently encouraged with a net to swim out of the test arena and back through the door, and the stimulus was removed. Successful trials (1) were when fish pecked the target dot within 30 s and unsuccessful (0) when fish did not peck the target dot). Each experimental session consisted of five trials, and 1–2 sessions were conducted per day. The position, size and order of presentation of the target dot was pseudo-randomised to ensure that each session consisted of a variety of difficult and easy target dots.

**Table 1.**
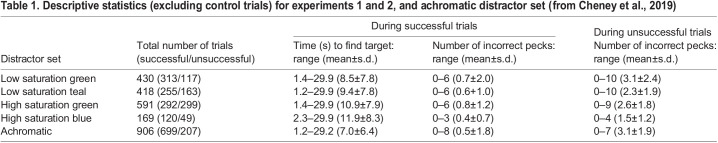
**Descriptive statistics (excluding control trials) for experiments 1 and 2, and achromatic distractor set (from** **Cheney et al., 2019****)**

### Visual modelling

The single cones of *R. aculeatus* contain a short-wavelength pigment (S; λ_max_=412 nm), while medium- (M; λ_max_=480 nm) and long-wavelength pigments (L; λ_max_=528 nm) are found in the two members of the double cone, which are used independently for trichromatic colour vision (Pignatelli et al., 2010; Cheney et al., 2013). Like many fish, *R. aculeatus* has a regular cone mosaic, with a single cone surrounded by four double cone members so that S, M and L cones occur at a ratio of 1:2:2 throughout the retina (Champ et al., 2014). Their cornea contains a yellow pigment; therefore, we adjusted photoreceptor spectral sensitivities (Cheney et al., 2013) with 50% transmittance data of the yellow cornea (Fig. S1; as per Cheney et al., 2019). We also performed visual modelling for our experimental stimuli without the yellow cornea and using absolute spectral sensitivities (as per Champ et al., 2016, Land, 1981; Green et al., 2022); however, this did not alter our overall conclusions.

We assumed that both members of the double cone contribute to luminance perception, as per previous studies in *R. aculeatus* (Mitchell et al., 2017; Newport et al., 2017). We used the added input of both double cone members (M+L) to calculate double cone quantum catch information (Green et al., 2022).

### Experimental stimuli

We used Ishihara-style stimuli (Fig. 2), which were created and printed as per Cheney et al. (2019). Stimuli comprised an array of distractor dots that varied in size and luminance (colour difference from each other Δ*S*<0.5). Distractor dots ranged from 2 to 16 mm in diameter (10 sizes) and were resolvable by the triggerfish at the experimental test distance (Cheney et al., 2019). A single target dot that differed from the distractors in chromaticity was randomly positioned and was of one of the three largest sizes (10, 12 or 16 mm in diameter) to ensure that the task was not too difficult (Cheney et al., 2019).

We selected the target and distractor colours by their locations in RNL space (Fig. 2). In brief, we determined colour distance between target and distractor dot colours using the log-linear trichromatic version of the RNL model (Vorobyev et al., 1998) and the function RNLmodel in the colourvision R package (Gawryszewski, 2018). A von Kries correction for light adaption was applied using the average spectral reflectance of the distractor dots and background paper (as per Cheney et al., 2019).

As there are no direct measurements of receptor noise in *R. aculeatus*, we initially estimated a standard deviation of the noise in a single receptor cell (σ) of 0.05, which has historically been chosen as a conservative measure of visual performance (as per Champ et al., 2016, Cheney et al., 2019), being considerably less sensitive than the human LWS cone system (Wyszecki and Stiles, 1982). The noise in each channel is then calculated from relative photoreceptor abundance, which is 1:2:2 (S:M:L) (Champ et al., 2014). We assumed noise in the L mechanism to be (equivalent contrast) *w*_L_=0.05, so noise in the S and M mechanisms were estimated to be *w*_S_=0.07 and *w*_M_=0.05, respectively, as per other studies of teleost fish (Champ et al., 2016; Cheney et al., 2019; Escobar-Camacho et al., 2019). A full description of modelling and equations is provided in the Supplementary Materials & Methods.

For each distractor set (location in colour space), we selected four sets of target colours, which radiated from the distractors in different directions (Fig. 2). Distractors varied in luminance to prevent fish locating the target dot using brightness, but did not vary in hue or saturation (difference between distractors was Δ*S*<0.5). We define colour sets that lie on lines extending radially from the centre as varying in saturation, whereas colour sets that change in angle around the centre vary in hue (Fig. 1). Spectral reflectance curves, quantum catches and Δ*S* values are provided in the data depository (Green et al., 2022).

### Experiment 1: Low saturation colours

In experiment 1, we tested thresholds for low saturation colours using two sets of distractors termed ‘low saturation green’ (Fig. 2C) and ‘low saturation teal’ (Fig. 2D). Both of these distractor sets were located Δ*S*=2 from the achromatic point. The low saturation green distractor set used five different distractor luminance values, and was used to test four different directions in colour space, named LG1–LG4 (13 target colours; Fig. 2C). A total of 430 test trials were conducted with each target colour presented a mean (±s.d.) of 4.3±0.6 times. The low saturation teal distractor used four different distractor luminance values, and target sets displayed on this distractor set were termed LT1–LT4 (15 target colours; Fig. 2D). A total of 418 test trials were conducted with each colour presented 4.4±1.0 times (mean±s.d.). Fish were presented with the low saturation green and teal distractor sets during the same experimental sessions in a randomised order.

### Experiment 2: High saturation colours

In experiment 2, we tested colours more saturated than those used in experiment 1, located Δ*S*=6 from the achromatic point and termed ‘high saturation green’ and ‘high saturation blue’. The high saturation green distractor set comprised five distractor dot luminance values and was used to test thresholds for four colour sets named HG1–HG4 (21 target colours; Fig. 2E). A total of 591 test trials were conducted with each colour presented 4.8±1.4 times (mean±s.d.). The high saturation blue distractor set contained five different distractor luminance values and was used to test colour discrimination for colour sets named HB1–HB4 (9 target colours; Fig. 2F). A total of 169 test trials were conducted with each colour presented 4.6±1.7 times (mean±s.d.). As for experiment 1, fish were presented with the high saturation green and blue distractor set during the same experimental sessions in a randomised order.

### Statistical analysis

**Fig. 3.**
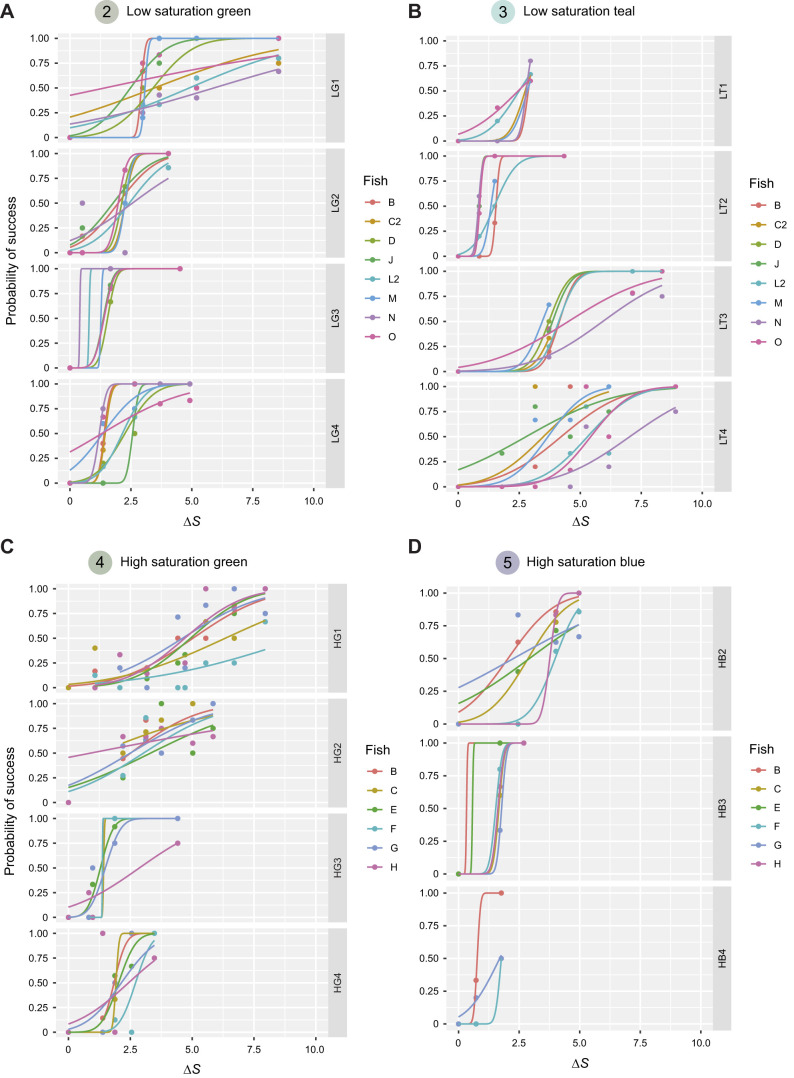
**Psychometric curves fitted for each fish for each target colour of increasing colour distance (Δ*S*) from the average chromaticity of the distractors.** (A) Low saturation green; (B) low saturation teal; (C) high saturation green; and (D) high saturation blue. Curves indicate the probability of successfully detecting each of the target colours within 30 s. Individual fish performance are shown by different coloured lines. Total number of trials are shown in Table 1.

We first calculated discrimination thresholds for each fish/colour set using the R package quickpsy (Linares and Lopez-Moliner, 2016) to produce cumulative normal psychometric curves. For each fish and each colour set, we examined how well our data fitted the psychometric curve generated using the deviance function, which was <14.3 (*P*>0.13; Fig. 3). We then used the function threshold to interpolate the 50% discrimination threshold (Δ*S*). We compared the colour thresholds for each fish using a linear mixed-effects model and the function lmer in R package lme4 (Bates et al., 2015) with thresholds as the response variable, colour direction as a fixed factor and fish ID as a random factor. We used lmertest (Kuznetsova et al., 2017) to produce *P*-values and the function glht in the package multcomp (Hothorn et al., 2008) to conduct *post hoc* paired comparisons between all directions on each distractor set. We corrected for multiple pairwise comparisons using the Bonferroni adjustment. We found no significant difference in colour discrimination thresholds between new and reused fish for any of the distractors tested (LMER, low saturation green: *t*_26_=−0.74, *P*=0.47; low saturation teal: *t*_5.54_=2.24, *P*=0.07; high saturation green: *t*_4_=0.86, *P*=0.44; high saturation blue: *t*_3.76_=−2.22, *P*=0.09­). Mean±s.d. thresholds for new and reused fish are presented in Table S1.

We could not calculate discrimination thresholds for some fish on some colour sets because their success rate for targets in a particular colour set did not reach 50% and their data curves could not be extrapolated. This was applicable to fish D and J for LT1 and fish D for LT4 (experiment 1), fish C, E and H for colour set HB4 (experiment 2) and all fish for colour set HB1.

For colour set HB1, we compared the data with the other three colour sets (HB2, HB3 and HB4) using a generalised linear mixed-effects model and the function glmer to compare the success rate of fish, rather than using the calculated discrimination threshold. We used a binomial distribution with 1=success (fish found the target dot within 30 s) and 0=unsuccessful (fish did not find the target dot). Colour distance (Δ*S*) from the average chromaticity of the distractors was included as a fixed factor.

To determine whether thresholds increased with more saturated colours, we used a linear model and the R function lm to compare thresholds for colour sets in a similar direction but on a more saturated distractor set. Descriptive statistics including the number of trials, average error rate, average time to locate the target dot, success rate and error rate are provided in Table 1.

## RESULTS

The experimental colour discrimination thresholds are given by the distance in the colour space (Fig. 2) for *R. aculeatus* relative to the value predicted by the RNL model. A threshold of 1 matches the model prediction, values <1 mean that the fish can detect smaller differences than predicted by the model and values >1 mean that performance is worse than predicted.

Colour discrimination thresholds varied depending on the direction and location in colour space. Colour thresholds near the achromatic point reported in Cheney et al. (2019) are presented here again for comparison with the current data and were lowest for the teal colour set (mean±s.d. Δ*S*=0.7±0.3), followed by the green (1.4±0.6), brown (2.3±0.4), blue (2.6±0.7) and pink colour sets (2.9±0.7) (Fig. 2A).

### Experiment 1: Low saturation colours

For the low saturation green distractor set, thresholds were lowest for the LG3 colour set (mean±s.d. Δ*S*=1.1±0.4), followed by the LG4 colour set (1.6±0.6) and the LG2 colour set (2.2±0.2), and highest in the LG1 colour set (3.4±1.4) (Fig. 2C). For the low saturation teal distractor set, discrimination thresholds were lowest for the LT2 colour set (1.1±0.3), followed by LT1 (2.7±0.2) and then LT3 (4.2±0.8). Thresholds were highest for the LT4 colour set (4.6±1.4) (Fig. 2D).

### Experiment 2: High saturation colours

For the high saturation green distractor set, thresholds were lowest for the HG3 colour set (1.6±0.6), followed by HG4 (2.2±0.3) and HG2 (2.2±1.0). Thresholds were highest for the HG1 colour set (5.8±1.8) (Fig. 2E). For the high saturation blue distractor set, thresholds were lowest for HB3 (1.3±0.6), followed by HB4 (1.4±0.6) and then HB2 (3.0±0.8). In the HB1 direction, no fish reached the 50% threshold, hence the discrimination threshold must have been greater than Δ*S*=4.6 (Fig. 2F).

As we found significant variation in discrimination thresholds across the colour space, we explored evidence for the hypotheses outlined in Fig. 1.

### Hypothesis 1: Noise in one or more photoreceptor channels exceeds that predicted by the relative number of the S, M and L receptors

**Fig. 4.**
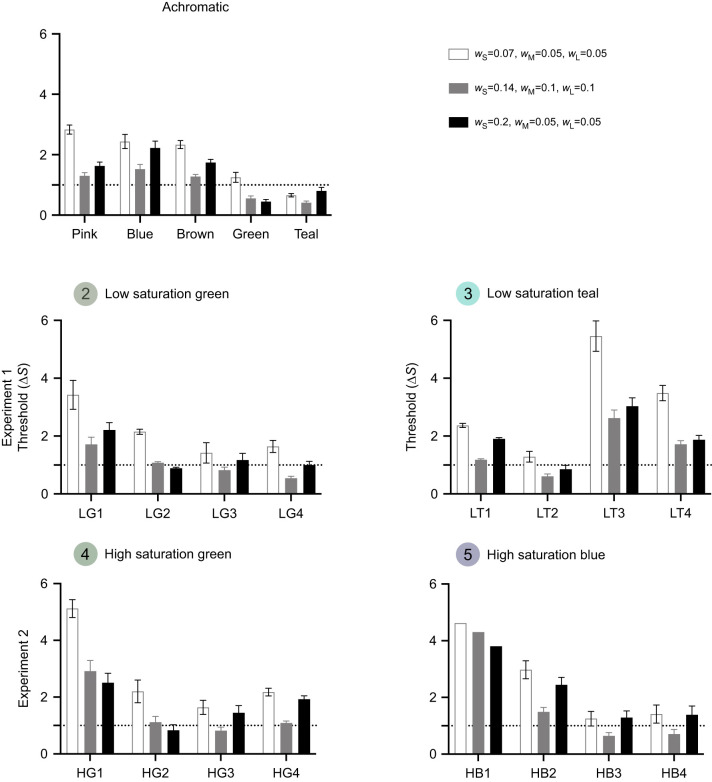
**Mean (±s.e.m.) colour thresholds for all five distractor sets when estimated based on relative cone abundance of 1:2:2 (S:M:L).** White bars assume a Weber fraction of 0.05 based on initial estimates, grey bars assume higher noise in all receptors and black bars assume higher noise only in the short-wavelength-sensitive receptor.

We initially estimated noise in the S, M and L receptor channels to be 0.07, 0.05 and 0.05, respectively (Vorobyev and Osorio, 1998), but our thresholds were high and were partially elongated along the *Y* axis (Figs 1 and 2), which could be consistent with increased standard deviation of noise in a single receptor cell (σ) or elevated noise in the S-cone mechanism. Therefore, we tested whether varying noise in each channel would produce behaviourally measured colour thresholds that were equivalent in each direction and fitted a circular loci of fixed radius (Δ*S*=1) (as described in Fig. 1A). First, we increased our standard deviation of the noise in a single receptor cell (σ) to 0.1 (to give *w*_S_=0.14, *w*_M_=0.1, *w*_L_=0.1), but thresholds varied between 0.41 and 2.92. Second, we increased the noise in the single cone compared with double cones (*w*_S_=0.20, *w*_M_=0.05, *w*_L_=0.05), because *R. aculeatus* has been shown to use single cones and both double cones members independently to achieve trichromatic vision (Pignatelli et al., 2010). Owing to the different anatomy of single cones, noise may not be determined by relative abundance alone. In this modelling scenario, thresholds varied between 0.80 and 3.03. Therefore, both of these corrections still left systematic variation in thresholds across colour space and could not explain our results (Fig. 4, Table S3).

### Hypothesis 2: Thresholds increase with distance from the achromatic point

To determine whether thresholds varied depending on the distance from the achromatic point, we compared thresholds for colour sets that changed in a similar direction through RNL space, but were located at different distances from the achromatic point (Fig. 2A). In our experiments, the adapting background consisted of the pooled achromatic colour of the paper visible between the distractor dots and the colour of the distractors, which comprised a similar proportion of the background area.

**Fig. 5.**
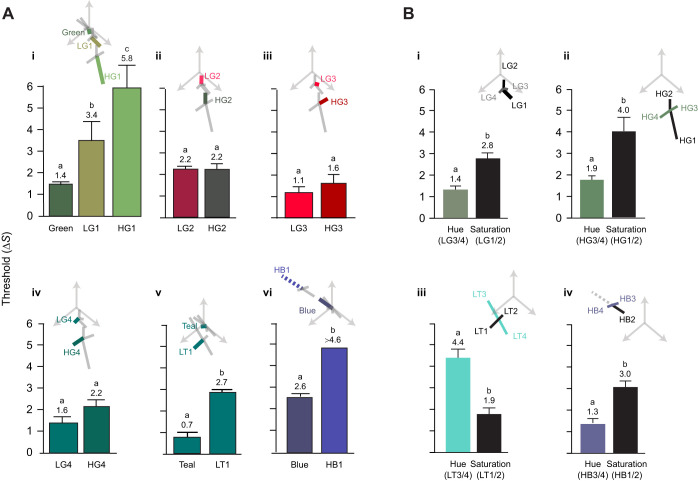
**Comparison of thresholds for colour sets with similar or opposing orientations in colour space.** (A) Thresholds for colour sets located at different distances from the adapted achromatic point. (B) Differences between colour sets which primarily change in hue and saturation. Thresholds are shown as bars indicating mean±s.e.m., as numerical values (mean) above bar and as bold scaled lines in colour space (inset). Different lowercase letters in the same graph indicate a significant difference (*P*<0.05). Remaining colour thresholds that were not compared are shown as grey lines. Thresholds for colour sets that primarily changed in hue for each distractor set were pooled, as were those that primarily changed in saturation. For example, in Bi, the saturation threshold is the pooled threshold of colour sets LG1 and LG2, whereas the hue threshold is the pooled average of LG3 and LG4.

In the green area of RNL space, thresholds for increasing saturation (i.e. colour sets green, LG1 and HG1) increased with distance from the achromatic point. Thresholds were lowest on the achromatic distractor set (green) and highest on the high saturation green distractor set (HG1). The three colour sets were also significantly different from each other (LG1 and green: *t*_14_=3.08, *P*=0.002; HG1 and green: *t*_12_=6.22, *P*<0.001; HG1 and LG1: *t*_12_=3.37, *P*=0.009; Fig. 5Ai). The remaining corresponding thresholds on the low saturation and high saturation green distractor sets were similar (LG2 and HG2: *t*_12_=0.15, *P*=0.88; LG3 and HG3: *t*_12_=0.47, *P*=0.65; LG4 and HG4: *t*_12_=1.98, *P*=0.071; Fig. 5Aii–iv).

Thresholds for colour sets that increased in saturation in the teal area of RNL space were larger for colours further from the achromatic point (thresholds were higher for colour set LT1 than for teal: *t*_12_=12.11, *P*<0.0001; Fig. 5Av). In the blue area of RNL space, we could not compare thresholds directly, because fish could not locate target dots from colour set HB1 on the high saturation blue distractor set. Therefore, we compared the likelihood of success of finding targets from colour set HB1 (on the high saturation blue distractor set) with that from colour set blue (on the achromatic distractor set), when accounting for colour distance from the distractors. This indicated that fish were less likely to locate targets on the high saturation blue distractor set, implying that thresholds are likely to be higher (*Z*=5.3, *P*<0.0001; Fig. 5Avi) further from the adapted achromatic point.

### Hypothesis 3: Thresholds for hue are smaller than those for saturation

We compared thresholds for colour sets that changed in hue with those that changed in saturation in the same part of RNL space. On each of the low and high saturation distractor sets, two colour sets primarily changed in saturation ­(suffix 1 or 2, e.g. LG1, LG2) and two primarily changed in hue (suffix 3 or 4, e.g. LG3, LG4). Saturation and hue directions were pooled for comparison. Thresholds for hue differed from those for saturation on all four distractor sets (Fig. 5Bi–iv). Thresholds were smaller, and therefore sensitivity was higher for hue compared with saturation in three out of four distractor sets: low saturation green (*t*_30_=4.19, *P*=0.0003), high saturation green (*t*_22_=3.06, *P*=0.006) and high saturation blue (*t*_8_=6.67, *P*=0.0001). However, for the low saturation teal distractor set, thresholds were higher for hue than saturation differences (*t*_28_=−6.00, *P*<0.0001).

## DISCUSSION

We measured colour discrimination thresholds for the fish *R. aculeatus* in four directions at four locations (16 directions) in the RNL colour space using Ishihara-style behavioural tests, and compared them with an additional five directions close to the achromatic point (Cheney et al., 2019). Thresholds varied between Δ*S*=0.69 and 5.79 depending on the direction and region of RNL space examined. Thresholds were closest to those predicted by the RNL model (Δ*S*=1, 18) at the achromatic point, and increased with distance from this location. In most cases, thresholds were also smaller, and sensitivity therefore higher, for hue than for saturation differences.

To understand the causes of these deviations from the RNL model predictions, we first examined whether our asymmetrical thresholds could be explained by the estimate of receptor noise (Fig. 1). Correction for different noise levels did not change the overall pattern of differences between colour directions and still left systematic variation in thresholds across colour space (Fig. 4, Table S3).

Our second hypothesis proposed that thresholds would increase for more saturated colours and/or increasing distance from the adapted achromatic point. Consistent with this hypothesis, we found that for green and blue directions, thresholds increased with distance from the achromatic point (Fig. 5), as they do in both humans (e.g. Danilova and Mollon, 2016, MacAdam, 1942) and birds (Silvasti et al., 2021; Lind, 2016). Likewise, thresholds for colours increasing in saturation (LG1, HG1 and HB1) were larger than those for decreasing saturation (i.e. LG2, HG2 and HB2) (Fig. 5). If it is assumed that the adaptation state of the visual system is fixed and colour differences are measured relative to the achromatic point, the Weber–Fechner law predicts an increase in thresholds with saturation and that the perceived change in a physical stimulus will be proportional to the magnitude of the perceived stimulus. The wide applicability of Weber's law implies that sensory systems encode relative stimulus magnitudes rather than absolute differences. For this reason we used the log-linear version of the RNL model (Vorobyev et al., 1998), which performs a logarithmic transformation of receptor light absorption. However, behavioural thresholds for highly saturated colours greatly exceeded our theoretical estimates of Δ*S*=1, which may be due to compressive nonlinearities (Lipetz, 1969) owing to photoreceptor or neural saturation, exceeding those predicted by the Weber–Fechner law (Endeman and Kamermans, 2010). Higher saturated colour sets were also located slightly further from the achromatic point. This may have contributed to the increased thresholds for more saturated colours, which has been demonstrated in research from both birds (Lind, 2016) and humans (Krauskopf and Gegenfurtner, 1992; Smith et al., 2000).

Our third hypothesis – that thresholds for hue would be smaller than for saturation (Fig. 1) – held for low and high saturation green, and high saturation blue distractor sets (Fig. 5vi,viii,x). However, on the low saturation teal distractor set, the difference was reversed (Fig. 5ix). Psychometric curves for hue discrimination were also usually steeper, with a more abrupt inflection point than those for saturation discrimination (Fig. 3). This may correspond with a distinct threshold at which point hue differences became detectable, compared with a more gradual function for saturation differences. Animals may benefit from enhanced sensitivity to hue compared with saturation because hue could provide a more reliable cue to object colour, being less affected by variation in shadows, or viewing geometry than either lightness or saturation.

Both hue and saturation differences are relevant to animal signalling, but enhanced sensitivity to differences in hue compared with saturation is well known in humans, termed the ‘super-importance of hue’ (Judd, 1968). There are few comparable studies for non-human species that can be tested in the same way, but Scholtyssek et al. (2016) found that thresholds for hue and saturation were consistent with RNL model predictions. Human thresholds for saturation discrimination are approximately twice those for hue discrimination, which is similar to three of the four threshold ratios observed here (saturation to hue ratio: low saturation green, 1.8; low saturation teal, 0.4; high saturation green, 1.84; high saturation blue, 2.9); however, we did not find this for low saturation teal (ratio=0.4). There is evidence that hue and saturation are processed separately in primate visual systems (Hanazawa et al., 2000), with hue represented by spatial location in the macaque visual area V4, whereas saturation may be coded by response magnitude (Li et al., 2014). Danilova and Mollon (2016) proposed that correlated neural noise within the visual system, possibly having a retinal origin, may explain why thresholds for saturation exceed those for hue.

Asymmetrical colour thresholds may also be attributed to opponent or higher level processing mechanisms within the neural retina at the eye or further up the visual pathway in the brain. Because opponent channels are unknown for most animals, the RNL model assumes unspecified opponent calculations (i.e. pairwise comparisons of all receptors). However, colour opponent mechanisms critically determine human colour thresholds (Hurvich and Jameson, 1957), and visual modelling indicates that the opponent channels used by an animal will expand and compress certain areas of colour space (Chittka, 1992). Colour thresholds may also be influenced by contextual factors such as the nature of the behavioral task employed (Dyer and Neumeyer, 2005; Avargues-Weber et al., 2010; Giurfa, 2004; Dyer and Chittka, 2004), categorical colour perception (Jones et al., 2001; Caves et al., 2018) and/or improved discrimination owing to sensory learning (Avargues-Weber et al., 2010; Dyer and Chittka, 2004). However, these processes are poorly understood in non-human animals and require further consideration.

In conclusion, noise in sensory systems sets discrimination thresholds, which – along with selective adaptation – have long been used to investigate sensory mechanisms. The RNL model is widely applied to predict the detectability and magnitude of colour differences for vision in non-human animals (e.g. Siddiqi et al., 2004; Santiago et al., 2020; Schaefer et al., 2008). However, our results highlight the need for a cautious interpretation of the RNL model and for further consideration of neural processing beyond the photoreceptors. We suggest that the threshold at which animals can detect the difference between two colours may be higher than model predictions for: (1) colours that differ from one another in saturation (rather than hue), and (2) highly saturated colours distant from the achromatic point in colour space.

## Supplementary Material

Supplementary information
