# Supplementary material for: Colour discrimination thresholds vary throughout colour space in a reef fish (Rhinecanthus aculeatus)
Source: J Exp Biol. 2022 Apr 11;225(7):jeb243533. doi: 10.1242/jeb.243533 (PMC9080749; doi:10.1242/jeb.243533)
Supplement: Supplementary information [file jexbio-225-243533-s1.pdf]

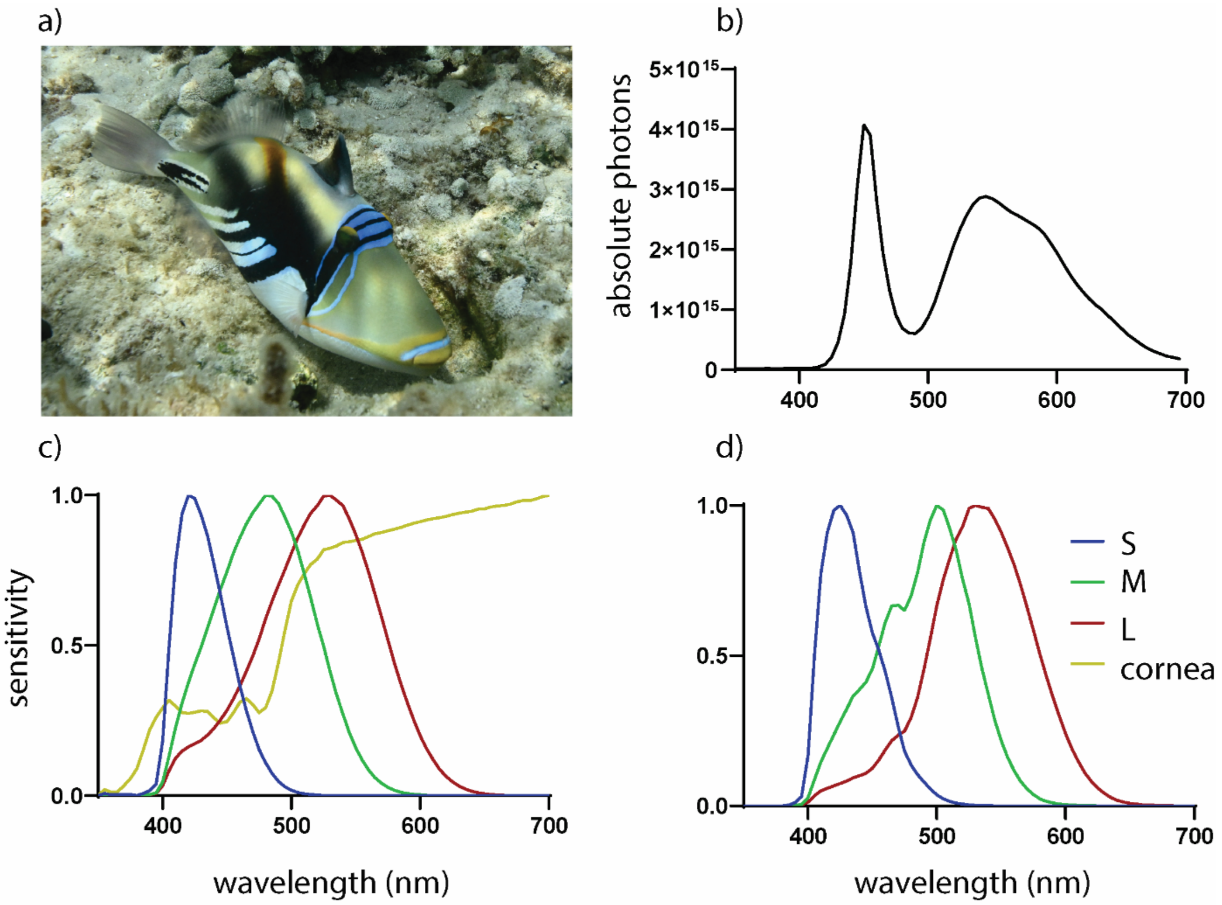

Fig. S1. a) The study species *Rhinecanthus aculeatus*; **b)** the downwelling illumination of the tanks (photons m^{-2} s^{-1} nm^{-1}); **c)** spectral sensitivities and yellow cornea transmission of R. aculeatus from **1**; **d)** spectral sensitivities adjusted by corneal filtering.

Supplementary Materials & Methods

Quantum Catch

The RNL model first determines the quantum catch (q) for the colored stimuli, in each of the three photoreceptor channels using the equation:

$$q_i = k_i \int_{300}^{700} I(\lambda)R(\lambda)C_i(\lambda)d\lambda \quad \textbf{[1]}$$

where I is the illuminant, R is the reflectance spectra of the printed color, C_i is the spectral sensitivity of receptor i, integration is from 300 to 700nm and k indicates the interval between measurements (here, 5nm). The photoreceptor spectral sensitivity curves (C_i) for *R. aculeatus* are from[1] combined with the 50% transmittance data for the yellow cornea from[2].

Journal of Experimental Biology • Supplementary information

Complementary colours were deemed isoluminant based on the normalised quantum catch of summed double cone members (table 1).

Von Kries Correction

The von Kries correction was applied because photoreceptors adapt to the light reflected off the background. To do this, the quantum catch of the entire visible scene was first calculated using:

$$Q_{Bi}(\lambda) = k \int_{300}^{700} I\,(\lambda) R_{back}(\lambda) C_i\,(\lambda) d\lambda \qquad \textbf{[2]}$$

where R_{back}, the reflectance spectra of the background. The von Kries correction was then used to normalise the quantum catch of the stimuli by the quantum catch of the entire visual scene, using the formula:

$$q_i = \frac{Q_i}{Q_{Bi}} \qquad \textbf{[3]}$$

The background area of the Ishihara-style stimuli was comprised of both the color of the distractor dots (50%) and the color of the paper (50%), visible between the distractors, therefore we used the average of these in the von Kris correction.

Receptor Noise Estimates

There are no direct measurements of receptor noise in *R. aculeatus*. Therefore noise was estimated based on relative photoreceptor abundance of each cone type as per [3], because it is assumed that the visual system improves the signal-to-noise ratio by averaging the signal of many affiliated photoreceptors [3]. Therefore, the equation used to estimate receptor noise (w) in channel i was:

$$w_i = \frac{\sigma_i}{\sqrt{n_i}} \qquad \textbf{[4]}$$

where n is the relative number of photoreceptors of type i in the retina (S:M:L; 1:2:2). We set noise in the LWS channel to 0.05, similar to other studies on teleost fish e.g. [4, 5, 6]. Therefore, noise in each channel was estimated to be 0.07, 0.05, 0.05 (S,M,L).

The Receptor Noise Limited Model

The distance between two colors *a* (the target color) and *b* (the color of the distractors) was calculated using the trichromatic version of the RNL model:

$$\Delta S = \sqrt{\frac{w_1^2(\Delta f_3 - \Delta f_2)^2 + w_2^2(\Delta f_3 - \Delta f_1)^2 + w_3^2(\Delta f_1 - \Delta f_2)^2}{(w_1 w_2)^2 + (w_1 w_3)^2 + (w_2 w_3)^2}} \qquad \textbf{[5]}$$

Where Δf_i is the difference in the log output of photoreceptor i for the reflectance spectrum of *a* and *b,* i.e.

$$\Delta f_i = \log\left(\frac{Q_{ai}}{Q_{bi}}\right) \qquad \textbf{[6]}$$

We used log photoreceptor outputs (i.e. the log-linear version of the RNL model) because most target colors were more than 1 ΔS from the distractors and the log-linear version is recommended for larger color distances.

Color Coordinates in RNL Space

Journal of Experimental Biology • Supplementary information

Color locations in RNL space were determined using the method described in [7], which is mathematically and functionally equivalent to that described in [8]. To use this approach, the coordinates of one color are set as the origin (0,0). The coordinates for the second color is then equivalent to

$$(x_2,y_2) = (d_{1,2}, 0) \quad \textbf{[7]}$$

where $d_{1,2}$ is the color distance determined using the RNL model between colors 1 and 2 (A and B).

The position of the third color (C) is then determined by finding the position in RNL space where the two distances, $d_{1,3}$ and $d_{2,3}$ are satisfied. This can be calculated using:

$$x_3=\frac{d_{1,3}^2- d_{2,3}^2+d_{1,2}^2}{2d_{1,2}} \quad \textbf{[8]}$$

$$y_3= \pm\sqrt{d_{1,3}^2 - x_3^2} \quad \textbf{[9]}$$

This equation can then be used to determine the location of all other colors relative to these three colors.

Journal of Experimental Biology • Supplementary information

Table S1. Colour discrimination thresholds for new and reused fish each colour set and colour direction. the lowest thresholds are shown in bold.

Colour Set	Colour Direction	New fish Mean + s.d.	Reused fish Mean + s.d.	Combined Mean + s.d.
**Low Saturation Green**	LG 1	**0.87 + 3.41 (n = 5)**	3.45 + 2.33 (n = 3)	3.43 + 1.41 (n = 8)
	LG 2	**2.15 + 0.26 (n = 5)**	**2.15 + 0.29 (n = 3)**	2.15 + 0.25 (n = 8)
	LG 3	1.17 + 0.28 (n = 5)	**0.98 + 0.50 (n = 3)**	1.09 + 0.37 (n = 8)
	LG 4	1.92 + 0.58 (n = 5)	**1.18 + 0.23 (n = 3)**	1.64 + 0.60 (n = 8)
**Low Saturation Teal**	LT 1	**2.69 + 0.17 (n = 3)**	2.70 + 0.22 (n = 3)	2.70 + 0.18 (n = 6)
	LT 2	1.13 + 0.32 (n = 4)	**1.08 + 0.40 (n = 3)**	1.11 + 0.33 (n = 7)
	LT 3	**3.83 + 0.26 (n = 5)**	4.86 + 0.94 (n = 3)	4.22 + 0.76 (n = 8)
	LT 4	**3.81 + 1.06 (n = 4)**	5.56 + 1.41 (n = 3)	4.56 + 1.45 (n = 7)
**High Saturation Green**	HG 1	6.23 + 2.72 (n = 3)	**5.43 + 0.78 (n = 3)**	5.83 + 1.84 (n = 6)
	HG 2	**2.14 + 1.11 (n = 3)**	2.33 + 1.09 (n = 3)	2.24 + 0.99 (n = 6)
	HG 3	1.91 + 0.83 (n = 3)	**1.37 + 0.07 (n = 3)**	1.64 + 0.61 (n = 6)
	HG 4	2.43 + 0.29 (n = 3)	**1.93 + 0.09 (n = 3)**	2.18 + 0.33 (n = 6)
**High Saturation Blue**	HB 2	3.34 + 0.94 (n = 3)	**2.61 + 0.49 (n = 3)**	2.98 + 0.79 (n = 6)
	HB 3	1.64 + 0.12 (n = 3)	**0.86 + 0.71 (n = 3)**	1.25 + 0.63 (n = 6)
	HB 4	1.73 + 0.05 (n = 2)	**0.77 (n = 1)**	1.41 + 0.56 (n = 3)

Journal of Experimental Biology • Supplementary information

Table S2. Mean absolute differences from DeltaS = 1 for each noise estimate / colour set. Green shading indicates thresholds < 1.

Colour Direction	Receptor Noise 0.07, 0.05, 0.05 (S,M,L) (as per manuscript)	Receptor Noise 0.14, 0.1, 0.1 (S,M,L)	Receptor Noise 0.2, 0.05, 0.05 (S,M,L)
LG1	2.43	0.71	1.21
LG2	1.15	0.08	0.11
LG3	0.10	0.18	0.17
LG4	0.64	0.46	0.00
LT1	1.37	0.18	0.90
LT2	0.20	0.39	0.15
LT3	2.50	1.62	2.03
LT4	4.20	0.72	0.87
HG1	4.80	1.92	1.51
HG2	1.20	0.12	0.17
HG3	0.64	0.18	0.45
HG4	1.18	0.09	0.93
HB2	2.20	0.49	1.44
HB3	0.25	0.36	0.29
HB4	0.41	0.29	0.39
Pink	1.42	0.30	0.63
Blue	1.60	0.45	1.22
Brown	1.33	0.28	0.74
Green	0.40	0.45	0.55
Teal	0.30	0.59	0.20
**Mean**	1.34	0.49	0.70
**St dev**	1.26	0.48	0.56

Journal of Experimental Biology • Supplementary information

References

1. Cheney KL, Newport C, McClure EC, Marshall NJ. Colour vision and response bias in a coral reef fish. *J Exp Biol* **216**, 2967-2973 (2013).

2. Siebeck UE, Marshall NJ. Ocular media transmission of coral reef fish - can coral reef fish see ultraviolet light? *Vision Res* **41**, 133-149 (2001).

3. Vorobyev M, Osorio D. Receptor noise as a determinant of colour thresholds. *P Roy Soc B-Biol Sci* **265**, 351-358 (1998).

4. Champ CM, Vorobyev M, Marshall NJ. Colour thresholds in a coral reef fish. *Roy Soc Open Sci* **3**, 160399 (2016).

5. Escobar-Camacho D, Taylor MA, Cheney KL, Green NF, Marshall NJ, Carleton KL. Color discrimination thresholds in a cichlid fish: *Metriaclima benetos*. *J Exp Biol* **222**, jeb201160 (2019).

6. Cheney KL*, et al.* An Ishihara-style test of animal colour vision. *J Exp Biol* **222**, jeb189787 (2019).

7. Pike TW. Preserving perceptual distances in chromaticity diagrams. *Behav Ecol* **23**, 723-728 (2012).

8. de Ibarra NH, Giurfa M, Vorobyev M. Detection of coloured patterns by honeybees through chromatic and achromatic cues. *J Comp Physiol A* **187**, 215-224 (2001).

Journal of Experimental Biology • Supplementary information
